# New perspectives on the ecology of tree structure and tree communities through terrestrial laser scanning

**DOI:** 10.1098/rsfs.2017.0052

**Published:** 2018-02-16

**Authors:** Yadvinder Malhi, Tobias Jackson, Lisa Patrick Bentley, Alvaro Lau, Alexander Shenkin, Martin Herold, Kim Calders, Harm Bartholomeus, Mathias I. Disney

**Affiliations:** 1 Environmental Change Institute, School of Geography and the Environment, University of Oxford, South Parks Road, Oxford, Oxon OX1 3QY, UK; 2 Department of Biology, Sonoma State University, 1801 East Cotati Avenue, Rohnert Park, CA 94928, USA; 3 Laboratory of Geo-Information Science and Remote Sensing, Wageningen University and Research, Droevendaalsesteeg 3, 6708 PB Wageningen, The Netherlands; 4 Center for International Forestry Research (CIFOR), Situ Gede, Sindang Barang, Bogor 16680, Indonesia; 5 Earth Observation, Climate and Optical Group, National Physical Laboratory, Hampton Road, Teddington, Middlesex TW11 0LW, UK; 6 Department of Geography, University College London, Gower Street, London WC1E 6BT, UK; 7 NERC National Centre for Earth Observation (NCEO)

**Keywords:** terrestrial laser scanning, tree architecture, metabolic scaling theory, wind speed, tree surface area, branching

## Abstract

Terrestrial laser scanning (TLS) opens up the possibility of describing the three-dimensional structures of trees in natural environments with unprecedented detail and accuracy. It is already being extensively applied to describe *how* ecosystem biomass and structure vary between sites, but can also facilitate major advances in developing and testing mechanistic theories of tree form and forest structure, thereby enabling us to understand *why* trees and forests have the biomass and three-dimensional structure they do. Here we focus on the ecological challenges and benefits of understanding tree form, and highlight some advances related to capturing and describing tree shape that are becoming possible with the advent of TLS. We present examples of ongoing work that applies, or could potentially apply, new TLS measurements to better understand the constraints on optimization of tree form. Theories of resource distribution networks, such as metabolic scaling theory, can be tested and further refined. TLS can also provide new approaches to the scaling of woody surface area and crown area, and thereby better quantify the metabolism of trees. Finally, we demonstrate how we can develop a more mechanistic understanding of the effects of avoidance of wind risk on tree form and maximum size. Over the next few years, TLS promises to deliver both major empirical and conceptual advances in the quantitative understanding of trees and tree-dominated ecosystems, leading to advances in understanding the ecology of why trees and ecosystems look and grow the way they do.

## 1. Introduction

Many who have gazed at the skeletal beauty of a bare, leafless tree on a winter's day have pondered why that tree takes the form it does, and why the form varies between, within and across tree species. Scientific interest in the structure of trees dates back to at least Leonardo da Vinci, who first observed that cross-sectional area of branches is preserved along branching orders within a tree (da Vinci's Rule [1]), and D'Arcy Thompson's seminal work *On growth and form* [2]. The concept of ‘tree architecture’ was advanced by Hallé & Oldeman [3] and later expanded upon by Hallé *et al*. [4], who proposed 23 distinctive descriptive models of tree form. The architectural form of a tree is a combination of both its genetic development programme and adaptive, semi-autonomous responses of individual branches to their local environment [5]. Nonetheless, despite the variety of architectural models and adaptive responses to environment, many trees have a form that is distinct and recognizable, which results in particular structural and ecophysiological characteristics. Tree form has important functional consequences too, in determining how fluxes of energy, water and carbon scale from leaf to tree to ecosystem [6], how trees can compete and pack together to form a forest stand, and how trees shape ecosystem structure, biomass and the habitat matrix for other species.

It is perhaps surprising, therefore, that tree structure and form are currently fairly neglected areas in ecology and plant ecophysiology, despite being of potential high theoretical and applied relevance in topics as diverse as remote sensing of canopy landscapes, taxonomy and phylogenetics, metabolic scaling theory, tree wind damage studies, whole-tree hydraulics, modelling of ecosystem functioning and carbon fluxes, and calculation of carbon stocks for climate change mitigation schemes. The primary reason for this neglect is that until now it has been difficult and time-consuming to quantify tree structure *in situ*, particularly for anything other than rather small trees (e.g. [7]). For example, studies on the full dynamic response of trees to wind have been confined to fewer than three painstakingly mapped three-dimensional tree models [8]. Moreover, what work there has been has tended to focus on low diversity systems consisting of one or two species [9,10]. This paucity of data has hindered development and testing of theory explicitly linking tree form parameters with physiological function. In the absence of sufficiently tested and established theory, most descriptions of tree form are based on purely empirical studies. For example, the biomass of tropical forests (a topic of high relevance in contemporary climate change mitigation and conservation policy) is largely estimated from entirely empirically allometric equations describing the form of a generic tropical tree based only on its trunk diameter and height [11], sometimes with some allowance of the influence of water stress [12], but with little recognition of the multiple environmental, taxonomic and biogeographical factors that may cause tree form to vary across the tropics.

**Figure 1.**
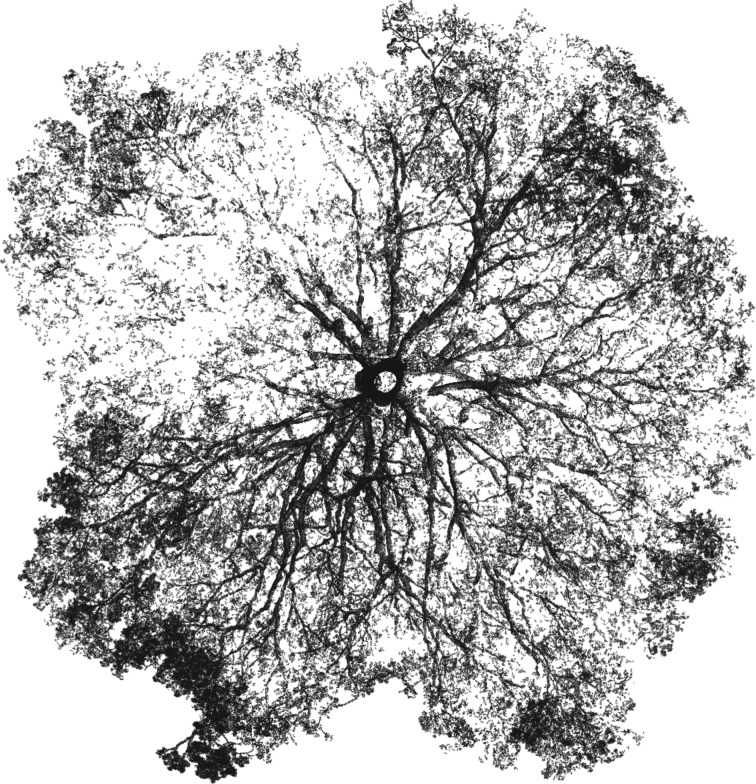
A top-down view of the architecture of a moabi tree (*Baillonella toxisperma*) in Gabon, illustrating the beauty in, and challenges to understanding, tree architecture and the potential of TLS approaches to deliver detailed quantitative descriptions of tree structure. The tree is 43 m tall, and the crown is 57 m across at its widest.

However, the study of tree architecture is now on the brink of a technology-driven revolution, with the advent of affordable and field-robust three-dimensional terrestrial laser scanning (TLS) technologies, also referred to as terrestrial lidar. TLS is a non-destructive remote-sensing technique for measuring distances [13]. TLS instruments emit a large number of laser pulses, typically tens to hundreds of thousands per second, in the visible or near-infrared part of the spectrum, which propagate hundreds of metres in the space surrounding the instrument. If a pulse hits an object, part of the energy is scattered back towards the sensor and triggers the recording of its distance and intensity. Knowing the direction of the emitted pulse, the position in a three-dimensional space is then recorded. These three-dimensional locations can be accurate to within a few mm over hundreds of metres, depending on instrument properties, and comprise a ‘point cloud’ describing the location of objects in three-dimensional space [14] (figures 1 and 7).

TLS instruments have been developed almost exclusively for surveying applications, particularly for urban, mining and geoscience, where mapping the position and orientation of large, hard surfaces is the aim (e.g. precise mapping of a building exterior or the interior surface of a mine). This has driven the development of instrument properties, particularly range (or power), beam divergence and wavelength. The instruments have not been developed specifically for ecological applications, which are a comparatively very small market. As a result, foresters, ecologists and remote sensing scientists have effectively adapted TLS to their needs by using commercially available instruments [15,16].

The obvious ecological relevance of the information content of three-dimensional point clouds has led to advances in data acquisition and analysis [17,18]. Firstly, field protocols have been developed to link many TLS scans across wider areas, via use of common registration targets, allowing capture of hectare-scale and greater point clouds [19]. Secondly, there has been a parallel development of new algorithms to convert point measurements into tree- and stand-scale properties of ecological interest, particularly size and shape of tree trunk and branch components [20–22], to separate leaf and wood components [23] and to generate vertical profiles of canopy density, which are critical to understanding and modelling canopy radiation regime [24].

As a result, TLS has become an automated, accurate, non-invasive, objective and repeatable option to assess tree stands, and potentially wider forest regions, with high detail. Its potential to assess tree structure has been proven over time [17,25]. Most recent TLS research has been concentrated on developing algorithms which provide a better understanding of the three-dimensional organization of tree structure, with ability to reconstruct and measure key attributes, such as tree location, stem density, canopy cover, above-ground biomass and diameter at breast height (DBH) with high accuracy from point cloud data [26–29]. This is providing many practical advances, including in more accurate estimation of volume and biomass [23,25], and descriptions of vegetation structure [30,31]. Work by [32,33] and others is the first step towards large-scale automated extraction of forest properties from TLS. Dassot *et al*. [34] provided an overview of TLS applications primarily aimed at forestry. They describe the state of the art at the time of forest structure estimation, particularly trunk shape and surface type, not just height and DBH. They also highlighted the difficulty of separating leaf and wood material in order to derive either wood or leaf properties separately. In the intervening period, the development and availability of higher precision, longer range TLS instruments has led to collection of much more detailed point clouds. This has led to increased focus on how to deploy TLS to collect much more detailed information on tree structure [19], and, in turn, on how to achieve topological descriptions of tree structure across all levels of branching. Progress in these areas has been somewhat symbiotic—developments in one have facilitated advances in the others, in turn.

Beyond description and quantification of tree structure and biomass, the rich information content of TLS data also opens up potential for testing and further development of ecological theory: not just describing what is found in terms of vegetation structure at a particular location, but understanding *why* it is found there and how that structure influences ecosystem function. In this paper, we focus on potential advances in our understanding of tree form and forest structure, but do note that these advances have many ramifications beyond trees alone, for example, in better describing and understanding animal habitat and use of space.

## 2. Understanding a tree

**Figure 2.**
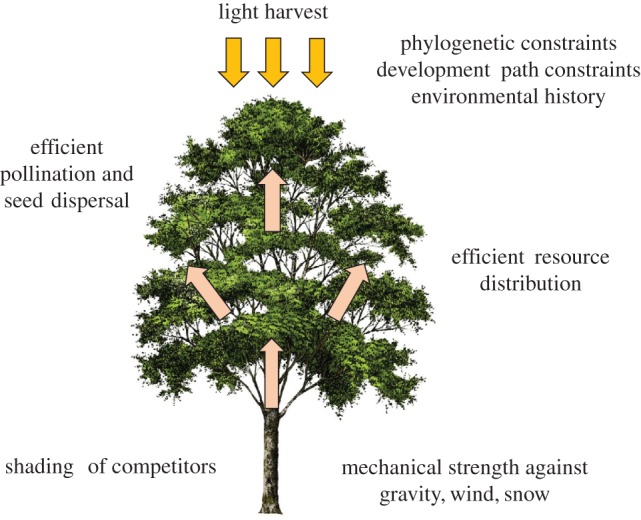
The architecture of a tree can be viewed as an attempt at an optimization of productivity, survival and reproduction subject to a number of requirements and constraints. (Online version in colour.)

The quantitative description of tree structure of large numbers of trees potentially enabled by TLS allow us to re-examine, further develop and test long-standing theories as to why trees have the shapes they do. A tree can be viewed (figure 2) as an attempt at optimization (of growth, survival and reproduction) subject to a number of requirements and constraints. These include (i) maximizing light capture and shading of competitors by maximizing vertical height, crown area and spatial arrangements between crowns [35]; (ii) maximizing the efficiency of resource distribution to and between the centres of metabolic activity (leaves, cambium and fine roots); (iii) minimizing risk of breakage or overturning from wind or buckling under gravity; (iv) minimizing water stress under gravity or drought conditions; (v) optimizing reproductive potential through pollination and seed dispersal; (vi) accommodating evolutionary constraints on development and growth pathways; and (vii) carrying legacies of past environmental history (e.g. past shade environment, wind damage). Not all trees or all environments will be subject to all these constraints. The solutions to this optimization determine the size and form (allometry) of trees, which end up strongly influencing the structure and habitat of woody ecosystems, the amount of biomass and carbon stored in the ecosystem, and the material flow of water and energy through the system.

In this paper, we present several examples of how TLS data are providing new data-rich approaches for understanding tree form in the context of these constraints.

## 3. Resource distribution and branching architecture

Branching architectures are pervasive throughout living systems, and their design is hypothesized to reflect trade-offs to access and fill three-dimensional space in order to transport resources with maximum efficiency and minimal cost [36]. In the context of trees, this leads to fractal-like structures that capture light, distribute resources (carbohydrates, water, nutrients) and provide support. Tree branching architecture is an essential link between individual leaf performance and whole-tree performance. In particular, West *et al.* [36] and Savage *et al.* [37] use metabolic scaling theory to argue that the overall metabolism of a tree (and its scaling with size) is intimately related to the properties of both its external branching architecture and its internal branching architecture (i.e. xylem vessels). In their strictest form these theories make specific predictions related to tree function based on the optimal external branching architecture of perfectly symmetrical and space-filling trees [37,38].

**Figure 3.**
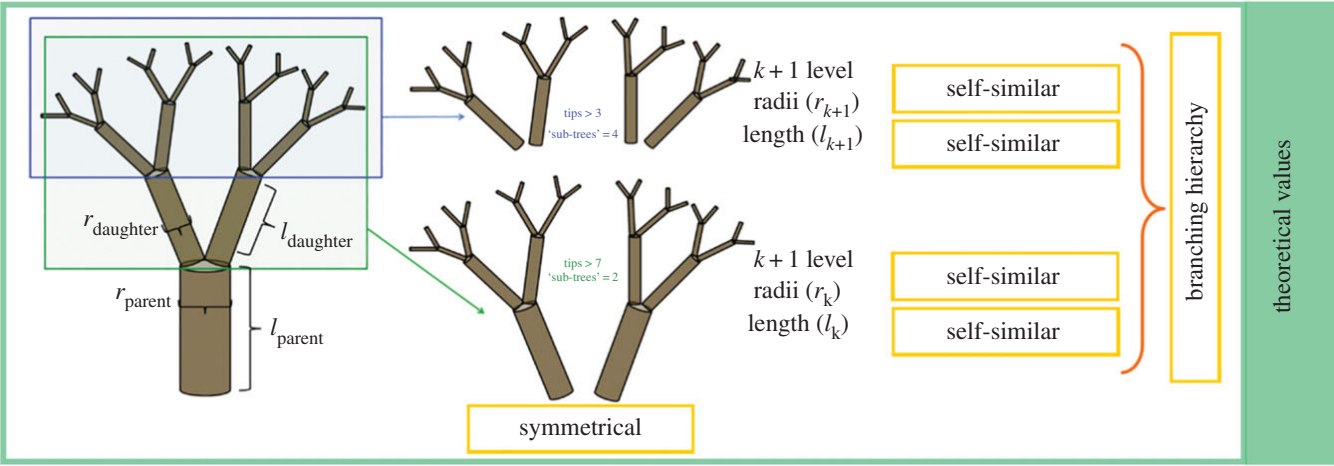
Branch scaling parameters for idealized symmetric trees. The branching ratio *n* is the number of daughter branches per parent branch (*n* = 2 in this example); the branch radius scaling parameter *a* = <*r_k_*_+1_/*r_k_*>, the ratio of the radii of daughter branches to those of their parent branches at each branch segment, averaged across a particular branching order; the branch length scaling parameter *b* = <*r_k_*_+1_/*r_k_*>, the ratio of the lengths of daughter branches to those of their parent branches at each branch segment, averaged across a particular branching order. (Online version in colour.)

In metabolic scaling theory, for idealized symmetric trees (figure 3), three key parameters currently describe branching structure: (i) branching ratio *n* (the number of daughter branches per parent branch), (ii) branch radius scaling, *a*, and (iii) length scaling ratio, *b* [38]. The radius and length scaling ratios relate to the conservation of cross-sectional area and the space-filling of volume. Using various assumptions related to network geometry [37–39], constant values can be given to these parameters across idealized trees where *n* = 2, *a* = ½ (Da Vinci's rule) and *b* = 1/3. The values of these parameters directly relate to the calculation of whole-tree metabolism of a tree of given mass.

However, most trees do not show an optimal external branching network. Self-similarity rarely holds true across all levels of tree branching, the majority of woody stems taper along stem length and trees exhibit asymmetric branching at branch-level [7,40,41]. As such, new theory is necessary [7] and is currently being developed for trees with asymmetric branching [42]. Moreover, some critics have pointed out that scaling of metabolic rates does depend not only on the potential resource uptake but also on availability of resources [43,44]. Resources, such as light or nutrients, are a limited resource in tropical forests and light competition is high due to strong light gradient within the canopy. Nevertheless, these approaches can serve as a point of departure for calculating scaling relationships for actual observed tree architectures.

The architecture of individual trees and their crowns scales up to shape the structure, metabolic function and dynamics of the entire forest stand, through competition for filling of space and capture of light [6,45]. It has been argued that ‘the forest is a tree’ [41], i.e. that scaling laws across trees in a forest have many parallels with scaling laws among the branches of an individual tree, and that the two are intimately connected through the tree architecture.

**Figure 4.**
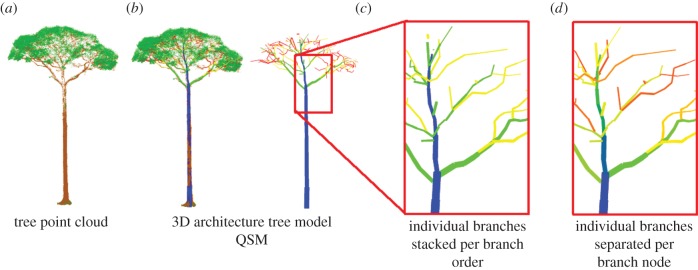
The application of TLS to extract and derive branching orders and tree architectural parameters.

The exquisite branching architecture of many trees is widely observed, but until recently, it has been difficult to describe this architecture in a robust and quantitative manner that spans scales from trunk to branch tip. This is an area where TLS has the potential to make major new contributions. Figure 4 shows how tree architectural parameters are extracted from point clouds. The tree point cloud is extracted from TLS scans (figure 4*a*), after leaves have been filtered out [23]. A quantitative structure model (QSM) is fitted to the tree to extract the tree form as a combination of multiple cylinders of varying length and diameter (figure 4*b*) [32]. From this QSM, the dimensions of individual branches can be calculated. Branches from the original QSM models are grouped by branch order. In figure 4*c*, the main stem does not split when a branch node appears and continues along to the top with the same branching order value (figure 4*c*). To describe a more detailed branch architecture, we can also separate individual branches at every branch node and reassign them unique values (figure 4*d*). The main stem now stops at the first branch node and unique value (colour) is assigned every time a split occurs.

As shown in figure 4, the unprecedented quality of three-dimensional structural data provided by TLS allows us to investigate old and new metrics to conceptualize tree architecture. One quantitative architectural metric that may prove useful is path fraction, which is the ratio of the mean path length (from tree base to branch tip) to maximum path length [46]. The path fraction provides a measure of the efficiency of the hydraulic system of the tree and has a maximum value of 1.0 for an idealized umbrella-shaped crown which prioritizes sun exposure and light capture but is structurally costly to build, but is lower for most tree crowns that have a mix of high sunlight and shaded environments. In addition to path fraction, we can also extract numerous other tree architecture parameters, such as branching angles, crown volume, crown asymmetry and the ratios of volume occupied by each branching order.

**Figure 5.**
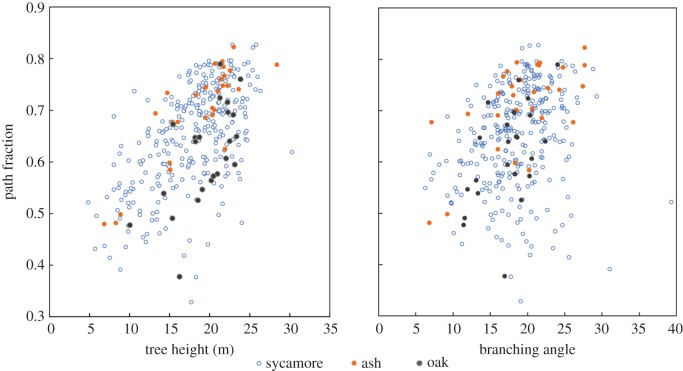
Path fraction against tree height and mean branching angle for trees of three species in temperate broadleaf woodland at Wytham Woods, Oxfordshire, UK: sycamore (*Acer pseudoplatanus*), European ash (*Fraxinus excelsior*) and English oak (*Quercus robur*). These architectural measures were extracted from Quantitative Structure Models (QSMs) derived from TLS point clouds.

Figure 5 gives an example of the relationships among path fraction, the mean opening angle between branches and tree height for three common tree species at Wytham Woods, Oxfordshire, UK. This demonstrates how efficiently multiple architectural parameters can be derived for hundreds of individual trees. It can be seen that path fraction tends to increase with height for all three species (i.e. trees become more umbrella-like as they reach canopy height to maximize access to sunlight). Among canopy trees, ash has a higher-path fraction than oak, whereas sycamore shows a much higher range in path fraction values. Remarkably, branching angle shows little correlation with path fraction or conservatism within species and appears an independent description parameter for tree architecture.

### 3.1. Surface area scaling and calculating tree respiration

Ecosystem ecologists have an interest in calculating tree respiration, or how much carbon is metabolized by the trees for their own growth and maintenance processes, and as a consequence is unavailable for biomass formation and consumption by other trophic levels. Around 50–70% of the carbon assimilated by photosynthesis in trees is used for autotrophic respiration and therefore unavailable for biomass production [47,48], and there is evidence that variation in autotrophic respiration may be particularly important in determining ecosystem carbon balance during extreme drought events [49]. There are challenges for measuring this for all organs, including leaves and fine roots, but a key challenge is the estimation for woody tissue, due to the problem of complex branching structure.

The most commonly used method to estimate total woody tissue respiration measures the amount of carbon dioxide emanating from the bark surface at one point on a tree's trunk, and applies allometric scaling assumptions to scale up from that point to the entire woody surface area of the tree [50,51]. There are a number of physiological assumptions in the scaling (e.g. around how the fraction and activity of metabolically active tissue varies through the tree architecture), but a key source of uncertainty is accurate assessment of the surface area of the tree. This is where TLS can provide significant insights and test existing allometric assumptions.

**Figure 6.**
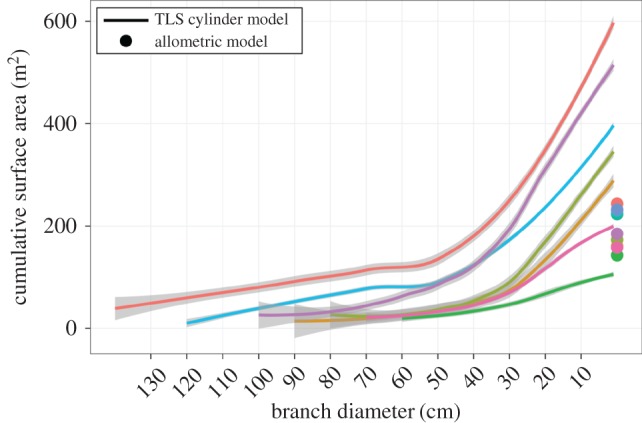
The application of TLS-derived models to show how cumulative above-ground woody surface area (over size classes of stems and branches) scales with DBH for seven tropical canopy forest trees in lowland Peruvian Amazonia. The dots (colours correspond to lines of the same colour) indicate the woody surface area calculated from a widely applied allometric equation for woody surface area from Chambers *et al.* [52].

Current TLS and model-fitting algorithms are capable of resolving tree branches as fine as a few centimetres in diameter. These models typically result in a set of cylinders arranged in space and connected via a topology. By summing the surface areas of these cylinders, we can derive estimates of total per-tree surface area, aggregated by branching order, diameter class or otherwise. These estimates are free from biological assumptions, are much faster to conduct than manual measurement, and can be conducted without felling the tree. Once surface areas have been summed, the point measurement of woody tissue respiration rate is multiplied by the total surface area to estimate total per-tree respiration rate (figure 6).

Figure 6 gives an example of how total tree woody surface area accumulates up through the branching architecture of seven lowland rainforest trees (of different species) in Tambopata, Peru. Smaller branches make a disproportionate contribution to total woody surface area; hence it is vital to both quantify this fine surface area and understand the metabolic activity of small branches and twigs. An allometric relationship widely applied to estimate woody surface area in tropical trees [52] tends to greatly underestimate woody surface area. There is also very large variation between tree species in surface area scaling, variation that is strongly related to differences in tree architectural form. TLS-acquired data such as these, when extended over thousands of trees, promise to enable development of a robust understanding on the relationship between tree form, woody surface area and tree metabolism.

### 3.2. Tree architecture and seed dispersal

Beyond resource acquisition and competition for space and light, a key plant functional role for which tree architecture can be important is reproduction and seed dispersal. For pollination, flowers need to be placed in positions visible and accessible to the relevant pollinators. For seed dispersal, there are likely to be correlations between dispersal strategy and tree architecture. To disperse tree seeds, a range of strategies can be employed from wind dispersal of small or winged seeds (favouring tree architectures that leave seeds prominent and exposed to the wind), bird dispersal (favouring prominently visible fruit), land mammal dispersal (allowing for larger seeds pods often requiring strong mechanical support), through to ballistic dispersal where seed pods ‘pop’ open and scatter to the ground (favouring extended branches spreading out from the core trunk). There has been little study of the relationships between tree form and seed dispersal syndrome, but this is an area ripe for study with the new availability of TLS data.

**Figure 7.**
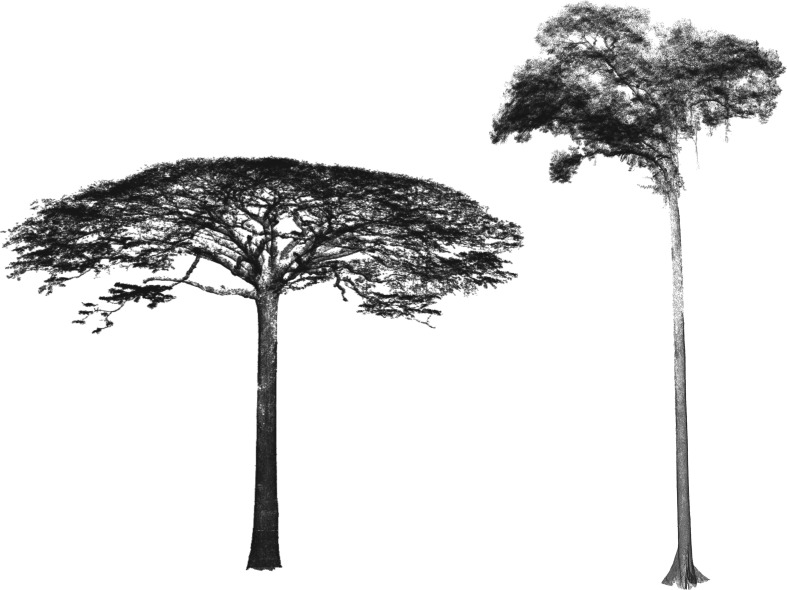
TLS-derived point clouds of two tropical giants: contrasting the structure of a moabi (*Baillonella toxisperma*; tree height 43 m, crown diameter 53 m) from Gabon (as seen from the top in figure 1) with a dipterocarp (species unknown; tree height 60 m crown diameter 30 m) from Sabah, Malaysian Borneo.

As an illustration, in figure 7 we compare TLS-collected point clouds of two tropical trees: a moabi (*Bailonella toxisperma*) from Lopé, Gabon, Central Africa, and a dipterocarp (species not yet identified) from Danum Valley, Sabah, Malaysian Borneo. The moabi produces large rounded fruit which are solely dispersed by forest elephants (*Loxodonta cyclotis*). The fruit drop from a great height, producing thuds in impact that announce their availability to infrasound-attuned elephants many kilometres away [53]. The broad stretching crown and massive branches of the moabi are well suited for dropping these heavy fruits from a great height and wide spread from the tree. By contrast, dipterocarps are a wind-dispersed tree family that dominates southeast Asian lowland rainforests that produce winged seeds that catch a gust and spin away from the mother tree. The crown of the dipterocarp is smaller with sparse clusters of leaves, enabling the tree to be taller for a given degree of mechanical strain and giving seeds easy access to wind gusts.

Differences in dispersal strategies and tree architectures matter beyond individual species. The dipterocarp-dominated, predominately wind-dispersed forests of southeast Asia are taller and different in structure from the (predominantly animal and ballistic dispersal) forests of Central Africa and Amazonia. Tree allometries relating tree diameter and height to biomass vary according to tree functional form, and more refined approaches to mapping biomass and structure across forests will need to take geographical variations of tree architecture into account.

### 3.3. Tree mechanics

The need to maintain mechanical safety is a fundamental constraint on tree architecture, and provides one of the limitations on tree height, which in turn limits biomass stocks in many forests worldwide. Understanding the role of wind in forest ecosystems has been most easily achieved in conifer plantations, where equal age and evenly spaced, single species forest structure coincides with extensive measurements of wind safety. Empirical and mechanistic modelling approaches have yielded good predictions of wind damage in this environment [54]. Pivato *et al.* [55] showed that conifers can be treated as simple pendula and so produced an accurate, large-scale model which accounts for the dynamics of tree sway. How much these approaches and insights can be applied to more complex forests with mixed ages, multiple species and broadleaf trees remains an outstanding question. Sellier *et al.* [56] used a sensitivity analysis to show that tree architecture is important in determining response to wind. Hence, outside conifer plantations, it is necessary to consider the three-dimensional structure of the tree to understand height limitation and predict wind damage.

**Figure 8.**
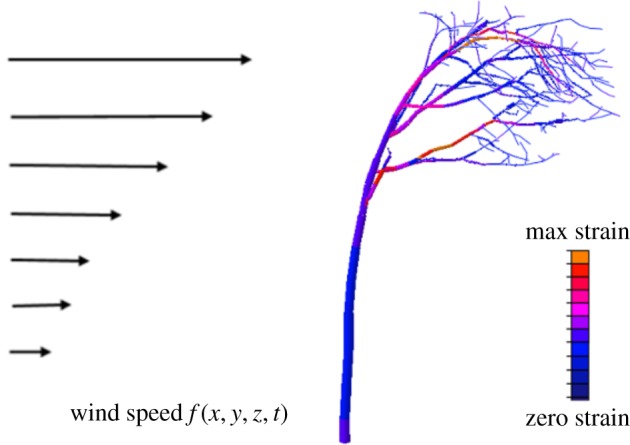
Overview of a tree-wind model using finite element analysis based on a QSM. An increasing wind force is applied to the model tree until the strain (a measure of deformation) reaches the breaking point.

The QSM approach allows the tree to be understood as a series of beams upon which the forces of wind and gravity act. Using finite-element mechanical modelling packages such as ABAQUS [57], it is possible to extract the significant sway modes and predict the critical wind speed at which a tree will break under a realistic wind forcing (figure 8). Preliminary results show that while conifers and other trees with a simple geometry have one or two significant sway modes, trees with large first order branches such as oaks have four to seven significant modes (Jackson *et al*. 2017, unpublished data). This means that their dynamic response to wind forcing is more complex and a more detailed understanding and description of tree architecture is required.

Previous work has predicted that trees tune multiple sway modes in order to pass potentially harmful kinetic energy efficiently between their trunk and branches, where it can be dissipated [58,59]. This damping by branching would increase the damping rate because trees ‘feel’ the force of wind on a two-dimensional projected area, but they dissipate the energy through a three-dimensional structure. TLS-derived cylinder models, as well as field measurements, allow the testing of this and other theories against hundreds of accurately mapped three-dimensional tree models.

**Figure 9.**
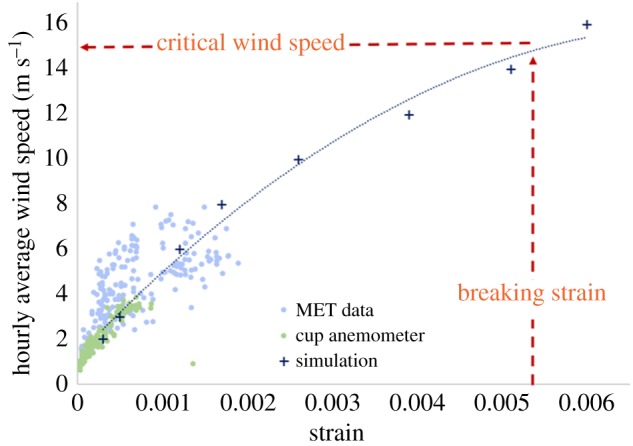
Critical wind speed estimate for a single tree calculated using the QSM-based approach with field data overlaid to give some estimate of the accuracy. The analysis suggests that an hourly average wind speed of around 15 m s^−1^ would result in a critical tree breaking strain. Data are for a silver birch tree (*Betula pendula*) in Wytham Woods, Oxfordshire, UK.

In order to investigate the effects of wind on the forest carbon cycle, we must consider direct wind damage and also height limitation. Preliminary data show that the critical wind speed at which a tree is predicted to break decreases with increasing tree height. This approach necessarily involves extrapolation past any available field data up to the breaking point of a tree (figure 9). Nevertheless, this approach would allow us to compare the calculated critical wind speeds of trees in a single plot and determine the plant growth and architecture strategies and trade-offs between trees which prioritize growth and those which display a more conservative wind damage avoidance strategy. Such approaches promise new mechanistic insights into one of the fundamental questions in forest ecology: what determines the heights of trees, and why does forest height show such geographical variation? As an example, Northern Borneo is known to have the tallest tropical forest canopies and individual trees in the world [60]. The tallest trees in the world are temperate conifers (e.g. the Californian coastal redwood, *Sequoia sempervirens*) which are likely limited by hydraulics (Koch *et al.* [60]), but the large variation in humid tropical broadleaf forest canopy height is poorly understood. Mean forest canopy heights in northern Borneo are around 45 m, compared to less than 30 m in Amazonia, even in regions with comparably high rainfall [61]. One plausible hypothesis for this disparity is that the maritime but non-cyclone prone wind regime of Borneo does not allow for extreme convective blowdown events that sometimes occur in the extremely continental wind regime of Amazonia. These rare extreme wind events may shape maximum sustainable tree height. The mechanistic modelling framework outlined above potentially allows us to directly test this hypothesis, for example by ‘exposing’ *in silico* tall Bornean trees to Amazonian wind regimes and seeing if and how frequently they would exceed critical wind speeds.

## 4. Outstanding challenges and future opportunities

We have illustrated above some of the potential that TLS provides in making fundamental advances in our understanding of the causes and consequences of variation in tree form. These analyses can be applied at wider scale, to understand how the interactions of individual tree form shape the structure and function of whole ecosystems, ranging from ecosystem energy availability and metabolism to the habitat and behaviour of animals. As the TLS revolution advances, this will emerge as an exciting and data-rich frontier in plant and ecosystem ecology.

To progress on this journey, however, a number of challenges still remain to be adequately addressed in the extraction and use of TLS data. These include (1) reliable extraction of higher order branching, (2) more robust fitting of cylinder models, and (3) reliable separation of woody from non-woody material (e.g. leaves) in the point cloud.

(1) *Separating trees from point clouds at speed and scale:* challenges remain in making accurate co-registration of many individual TLS point clouds much more rapid and ideally automated, and then also in automating extraction and processing of individual trees from these large point clouds. This would also make TLS data collection and extraction much more widely available beyond specialist research groups. Mobile laser scanning developments have seen progress in the first area. TLS instrument manufacturers have developed automated, targetless registration processes, particularly for mobile mapping applications (e.g. Riegl and 3Dlasermapping). These can work well, but typically only in environments with large, hard, regular surfaces such as built environments or supported with a strong GNSS/GPS signal. Work remains for them to be usable in forest environments dominated by many small, soft and irregular targets and under closed canopies. Tree extraction is also progressing rapidly. This process still generally requires some manual input, but as for the co-registration, increased interest in these applications is driving rapid development of full automation [62].
(2) *Extracting high order branching reliably:* owing to vegetation occlusion, the point cloud point density differs at different heights [19]. Point density is highest in the lower parts of the canopy (where tree branch structure is also typically larger), allowing three-dimensional reconstruction algorithms to model the point cloud very accurately. The point density becomes lower in the canopy, and branches are often occluded. The beam divergence of the laser leads to a larger pulse footprint with increased range, meaning that smaller objects in the top of the canopy cannot be resolved. This directly affects the way reconstruction algorithms can follow branching structure, tending to reduce the accuracy of three-dimensional cylinder models with height. This is relatively unimportant for applications focused on total volume/biomass, but more so for those exploring branch size distributions.
(3) *Fitting cylinder models:* fitting a cylinder model to a point cloud is a problem with many possible solutions. In practice, it is found that that small curves and changes of direction in branch structure are often best fit by multiple, short cylinders. From a mechanical or architectural point of view this is problematic, because neighbouring short cylinders may often point in slightly different directions. This can add surface area and reduces the mechanical viability of the structure. Simplification of the cylinder model by averaging together neighbouring cylinders provides a robust solution to this problem. Increasing amounts of accurate TLS data are enabling fitting models to be improved, and new approaches to be developed [33].
(4) *Separation of leaves from wood:* leaves are challenging to separate from wood in the TLS point cloud. This affects the accuracy of the retrieved structural models, and any resulting (woody) biomass and architecture calculations. This also has implications for tree mechanics modelling. The increase in sail area due to leaves has a strong effect on the response of a tree to wind forcing. But there are promising advances in extracting leaves, using a combination of methods to exploit differences in the return intensity of the signal, and using *a priori* assumptions of how leaves and branches are co-located in three-dimensional space [23].

## 5. Conclusion

New TLS methods allow for the explicit capture and mathematical representation of tree from in exquisite detail. Recent progress in the handling and processing of vast amounts of point cloud data allows for automatic extraction of tree architecture for large sample sizes. These advances are already enabling the development and improvement of estimates of tree allometry and forest structure [21,23,63]. Beyond these practical applications, these advances allow the development and, crucially, testing of new theory related to tree form, tree mechanics and forest structure. These are disciplines that remain relatively unexplored for diverse broadleaf forests. The opening of this new frontier has been driven by technological and computational developments. Further technological progress is likely in coming years, such as the deployment of laser scanning with multiple, vegetation-sensitive wavelengths to monitor plant water status in three dimensions [52], and improved separation of leaf biomass from woody biomass [64]. For ecologists an exciting challenge is to integrate this flood of new and extremely detailed data with existing theory on tree and forest structure. This will require a combination of analytical methods with explicit simulation of growth environment and biomechanics, to describe and understand how tree form varies with phylogeny, development stage and environment. This offers the promise of predictive and robustly tested theory of tree form and forest structure, a valuable toolkit that may allow us to understand why different forests have the structure they do, and how ecosystem structure and biomass may change in a world of multiple anthropogenic influences on forest ecosystems.

## Data accessibility

This article has no additional data.
